# Book Review

**Published:** 1937

**Authors:** 


### Reviews of Books

**A Text-Book of the Practice of Medicine.** Edited by F. W. Price, M.D., C.M., F.R.C.P., F.R.S.E. Fifth Edition. Pp. xli., 2,038. London : Oxford University Press (Humphrey Milford). 1937. Price 36s. (printed on India paper, 45s.).—Judging from the requests which reach the librarian, a new edition of Price's *Text-book of Medicine* must be a great event in the life of the average medical student. A comparatively brief glance at the fifth edition will suffice to show that this great popularity is truly well deserved. Price's *Medicine* is probably the most successful example of the symposium type of text-book in which each subject is dealt with by its own particular specialists, and it is a very worthy product of the London School of Medicine. For the student it is more than a mere text-book—in fact, it might almost be called an encyclopedia, since it not only covers the whole of general medicine, but in addition deals fully with tropical medicine, diseases of the skin and psychological medicine. The scope of the book may be illustrated by the fact that sections added to the new edition range from chiufa to glycogen disease and from leuco-erythroblastic anæmia to electrical injuries. Many sections have been rewritten, a fresh classification of Bright's disease is introduced, and the nomenclature of the anæmias has been altered. With the continual addition of new matter, and the incorporation of fresh advances, the book is bound to increase in size despite the most vigorous pruning, and the fifth edition sees a new attempt to cope with this. An India paper edition has been published in addition to the ordinary copy. Thus to those who can afford it there is available a complete edition much reduced in bulk and weight. The text in both is clear, although in the ordinary edition the thin type of paper used tends to allow the print to show through from the opposite page. It is difficult to see how this can be avoided without making two volumes. The illustrations are clear and the index full and adequate.

**Minor Maladies and their Treatment.** By L. WILLIAMS, M.D. Seventh Edition. Pp. xiii., 439. London: Baillière, Tindall and Cox. 1937. Price 10s. 6d.—This little book, which has run into many editions since its inception in 1906, is written for the general practitioner who starts straight from hospital, with very little idea of how to treat the common minor medical maladies which surround him. Dr. Williams mentions in the introduction that the book is egotistical and, in parts, heterodox. Reading the book, one finds it very easy to agree with him. Many of his methods are out of date, intestinal toxæmia features very frequently, and a "gouty diathesis" is the basis of many ills. His conclusions, in many cases, lack either scientific or therapeutic proof, and many of his prescriptions tend towards polypharmacy. There are helpful chapters on general health, certification of lunatics and dietetics, but one feels that a chapter on the recognition of the more common neuroses should be included in any book of this nature. It contains, however, many glimpses of wisdom and is an eminently readable and, in parts, rather amusing book.

**Treatment in General Practice.** By several authors. (Reprinted from the *British Medical Journal.*) Pp. x., 250. London: H. K. Lewis & Co. 1936. Price 8s. 6d.—This book, a (delayed) republication of a series in the *British Medical Journal*, 1934–1935, consists of thirty-six articles by different authors on respiratory, infective, and vascular disorders, the whole designed to present in unison "a panorama of current therapeutics." Present clinical preoccupation and approach are shown by the place of coronary thrombosis, thyrotoxic heart, and new growth in the lungs amongst the older orders—pneumonia, septicæmia, rheumatic carditis. Practitioners of early or of long experience will find guidance or confirmation in what to adopt or discard in doubtful or critical hours. The editors are aware that the art of medicine does not stand still, and that day by day the laboratories make new gifts to the bedside. The book opens with Lord Horder's observations on influenza, and concludes with electrocardiography (Dr. A. Hope Gosse), "the writing which is still an unknown language to many."

**Failure of the Heart and Circulation.** Edited by T. EAST, D.M., F.R.C.P. Pp. vii., 130. Illustrated. London: John Bale, Sons and Curnow Ltd. 1937. Price 2s. 6d.—In this contribution to the Pocket Monograph series Dr. East has attempted to explain in a simple way what happens in the circulation when the heart fails. He has succeeded admirably. The book can be most heartily recommended both to busy practitioners and to students as a companion to their text-book of general medicine. It summarizes in a most lucid and interesting manner the modern views on failure of the heart. Symptoms are discussed and explained on a physiological basis, and we are also shown how to recognize the warning sign of impending cardiac failure. A large chapter is devoted to treatment, which is dealt with on similar physiological principles. The text is clear, and there is a good index.

**Diseases of the Heart.** By Sir THOMAS LEWIS, C.B.E., F.R.S., M.D., D.Sc., LL.D., F.R.C.P. Second Edition. Pp. xx., 297. London: Macmillan and Co. Ltd. 1937. Price 12s. 6d.—In the second edition of his invaluable exposition of diseases of the heart Sir Thomas Lewis has revised the whole text and incorporated the relevant recent advances in our knowledge. This book presents in simple and understandable language the essential facts of cardiology, and throughout the keynote has been simplicity. Ruthless pruning has stripped the subject of all the needless detail and trite phrases with which medicine is encumbered at the moment. Emphasis has been laid on easily appreciated physical signs, and the dominant importance of the patient's symptoms is stressed throughout. Representing as it does an outline of the clinical teaching, which has hitherto been the exclusive privilege of students of University College Hospital, it can be wholeheartedly recommended to all those students and practitioners who have been less fortunate.

**Infants in Health and Sickness.** By R. E. STEEN, M.D., F.R.C.P.I. Pp. xi., 127. Illustrated. London: Oxford University Press. 1937. Price 5s.—All who are conversant with the infant section in Tweedy's *Practical Obstetrics* will welcome the appearance of this excellent synopsis under a separate cover. The work deals mainly with infant feeding and digestive disorders, and is designed for general practitioners and students, and for all members of the nursing profession concerned in the care of infants during the first year of life. In presenting a difficult subject clearly and concisely in little more than 100 pages the author has achieved what some have failed to do in two or three times the space. The book is, in fact, almost a miracle of compression, and should prove of extreme value for quick reference particularly to the newly-qualified doctor faced for the first time with the many practical and sometimes urgent problems connected with this branch of his work. The book concludes with a useful list of diet sheets and a table showing the composition of various proprietary foods.

**Chronic Streptococcal Infection as a Disease.** By J. D. HINDLEY-SMITH, M.A., M.R.C.S., L.R.C.P. Pp. x., 44. London : H. K. Lewis & Co. Ltd. 1937. Price 3s. 6d.—In this small volume of fifty pages the author has summarized his views on the " disease entity of chronic streptococcal toxæmia," a subject previously dealt with by him at greater length in a volume which was reviewed in these columns (Vol. LIII., No. 199, 1936). He has added little to his conception of the continuity of chronic streptococcal infection from childhood to middle age. His diagnostic proof of the part played by the streptococci is still the " streptococcal index " of throat swab cultures, and his main line of treatment is still subcutaneous autogenous vaccines, to which he now adds " spray " and " oral " administration of streptococcal suspensions. He justifies this use of different types of vaccines by unorthodox and somewhat contradictory hypotheses, but his main purpose of attracting attention to the possibility of avoiding many of the chronic ills of middle and old age by the eradication of streptococcal infections in childhood is still the manifest and commendable basis on which this volume should be judged.

**Reports on Chronic Rheumatic Diseases, No. 3.** Edited by C. W. BUCKLEY, M.D., F.R.C.P. Pp. x., 131. London : H. K. Lewis & Co. Ltd. 1937. Price 10s. 6d.—This third volume of the Report is, if anything, better than the two earlier numbers. The problem of chronic rheumatic diseases is discussed authoritatively from a number of standpoints. Several of the articles are in the nature of stock-taking of our knowledge and attitude towards certain therapeutic measures, *e.g.* vaccines, sympathectomy, X-Ray therapy, and chrysotherapy, and these will be found particularly valuable ; so also will the synopsis of recent American investigations by the Secretary of the American Committee for the Control of Rheumatism. Among the many other smaller articles and reviews should be mentioned the critical commentaries on " Vitamin Deficiency and Chronic Rheumatism," " The Place of Sodium Salicylate in the Treatment of Gout," and "Still's Disease." The whole volume is packed with valuable and interesting material and there is no doubt that the maintenance of the high standard of excellence of these Reports will ensure an increasing demand for them and for further numbers of the series as they appear.

**Medico-Legal Aspects of the Ruxton Case.** By J. GLAISTER, M.D., D.Sc., and J. C. BRASH, M.D., F.R.C.S.E. Pp. xvi., 284. Illustrated. Edinburgh: E. & S. Livingstone. 1937. Price 21s.—The contemporary press is always anxious to give full publicity to the medical aspect of crimes that attract nation-wide interest, but to the medical reader the information appears frequently distorted, and essential facts in the chain of evidence are lacking. It is therefore of considerable interest to read the whole clinical details of the Ruxton case, and to find them set out with complete lucidity. The authors have given the scientific evidence in full detail and illustrated with excellent, if somewhat gruesome, photographs. One of the outstanding features of the case is the diverse specialities involved; radiology, dentistry, chemistry, hæmatology and many other aspects of medical science all played a part in the chain of circumstantial evidence that led up to Ruxton's conviction. The book is a mine of information on the utilization of modern medico-legal methods and should prove of real interest to many readers. The concluding chapter is devoted to Mr. Justice Singleton's summing-up of the scientific evidence, and in view of the importance of the legal approach to scientific evidence, might well have been given in greater detail. Altogether a most interesting book that well repays perusal, although not to be recommended for reading last thing at night; it is too like the Chamber of Horrors.

**The Sanitary Inspector's Handbook.** By H. H. CLAY. Third Edition. Pp. xxii., 480. Illustrated. London: H. K. Lewis & Co. Ltd. 1937. Price 16s. 6d.—It was anticipated, in view of the new legislation which has recently come into force, namely the Public Health Act, 1936, the Housing Act, 1936, the Public Health (London) Act, 1936, and other Acts and Regulations connected with public health, that a new edition of this very well-written and useful book might be expected. The book is right up to date—all the amendments and modifications of the procedure rendered necessary by the new legislation are very clearly described in detail. The format is adhered to, the same excellent arrangement of chapters, each concise in form but containing a wealth of information covering every possible aspect of the particular section. The epitome of the legislative clauses which commence each chapter is couched in simple and easily readable language and forms an excellent introduction to the subject-matter which follows. There are several additional illustrations, that showing a plan of the drainage system of a dwelling-house being a fine example of reproductive detail. There are over a hundred more pages in the new edition and each chapter has been revised and enlarged. The author has in this practical and concise handbook produced a volume which will be regarded not only as a standard work for students, but a reference for various classes of public health officials. We can recommend this new edition, crammed with technical information, well written by one who is a recognized authority on his subject, not only to sanitary inspectors but to all officers engaged in the public health service.

**The Scientific basis of Physical Education.** By F. W. W. GRIFFIN, M.D. Pp. viii., 203. London: Oxford University Press (Humphrey Milford). 1937. Price 7s. 6d.—The general public is, at the present time, being made very health-conscious, what with the State campaign for national fitness and the British Medical Association's proposals ; national playing fields and Leagues of "Health and Beauty," and so on. Dr. Griffin's book sets out to assist the medical man who may be called in consultation by organizers of physical education and general "fitness campaigns." It is largely a summary of the scientific knowledge now available, and quotations abound, especially from Samson Wright's *Applied Physiology*. There is also a very comprehensive bibliography of over sixteen pages out of the two hundred which make up the book. In the last chapter an attempt is made to designate physical types in the same race, for determining the suitable form of education. The book is undoubtedly sound and is presented in the publishers' usual efficient manner.

**Post-graduate Surgery.** Edited by R. MAINGOT, F.R.C.S. (Three volumes.) Pp. xii., 2,012. Illustrated. London : Medical Publications Ltd. 1937. Price 70s.—This is truly a notable publication. The first volume appeared about a year ago, and now the third and last has seen the light of day. It may be explained, briefly, that nearly all the many contributors are London surgeons, and most of them young men ; that in the general plan of the book it is taken for granted that the reader has a fair knowledge of surgery as it is set forth in the classical text-books, but that he wishes to be put in touch with the new classifications, new methods of diagnosis, and new methods of treatment. It is far more than a text-book of operative surgery. The first volume deals with anæsthesia, diseases of the abdomen and rectum, X-ray diagnosis, and radium. The second volume includes the bulk of ordinary surgery and gynæcology. In the third, diseases of the eye, ear, nose, throat, œsophagus, mouth, and teeth find a place, together with obstetrics, venereal diseases, plastic surgery, orthopædics, and a few general surgical subjects such as diseases of the cardio-vascular and lymphatic systems, and useful articles on small special topics such as blood-drip transfusion, cisternal puncture, pilonidal sinus, burns, and treatment by snake venom. The book is of very great value ; we know nothing else that fulfils the same purpose. It is excellently illustrated, and the print is unusually large and clear. Where all is so good, it is difficult to single out any part for special praise, but we personally read with great interest and profit the section on the Urinary System and Male Genital Organs.

**Physical Signs in Clinical Surgery.** By HAMILTON BAILEY, F.R.C.S. Sixth Edition. Pp. xii., 284. Illustrated. Bristol : John Wright & Sons, Ltd. 1937. Price 21s.—This is a wonderful little book, and we are not at all surprised that six editions have been called for in ten years. It very rightly directs attention to the personal examination of the patient by the doctor or medical student. In these days when there is such a tendency for the clinician to shirk his work and throw the responsibility for a diagnosis on to the pathologist, or the hæmatologist, or the radiologist, or the bio-chemist, this is a very salutary admonition. Though mainly written for the medical student, the book is vastly instructive for the medical practitioner. For one thing, it teaches how to examine the patients without hurting them. For instance, we are told, in the case of a child with suspected intussusception, to begin by palpating the abdomen, with a warm hand, under the bed-clothes. Some of the diagnostic signs will probably be fresh to the professional surgeon also. To locate an arterial embolus, we are told to occlude the limb high up with a band, and listen with a stethoscope over the artery ; when the band is let go, the booming sound can be heard down to the embolus, where it stops suddenly. Or, draw the prongs of a fork down the limb ; vasomotor stripes appear, which fade at the level where the circulation is interrupted. To distinguish between intussusception and Henoch's Purpura, apply a band for three minutes to a limb fairly tightly ; petechiae will appear below it. Valuable as the text is, the main value of the book lies in its 358 illustrations, all good, and some very good indeed. Merely to glance through these is a valuable education. If it were not for them, one might think a guinea a large price for a book on so limited a subject, but it is money well spent. To learn to diagnose is well worth our money and our pains.

**The Principles and Practice of Rectal Surgery.** By W. B. GABRIEL, M.S., F.R.C.S. Second Edition. Pp. ix., 363, Illustrated. London: H. K. Lewis & Co. Ltd. 1937. Price 28s.—The second edition of this book, some five years after the first, shows many useful additions to the first volume, as well as thorough revision. The work throughout conveys the principles associated with the St. Mark's Hospital School, and this alone would stamp its authoritativeness. Mr. Gabriel has written in a very clear and reasoned manner on all the common rectal affections. He gives full details of all his methods, and, even better, full illustrations wherever feasible. The book contains over 170 illustrations in its 350 pages of text. This makes the book an excellent manual for the general practitioner who is prepared to undertake some minor rectal surgery, as well as a reliable book of reference for the practising surgeon, and a concise account for the student. There is, indeed, little to criticize, and this review must content itself with a description of the work. A chapter on surgical anatomy, written in conjunction with Mr. O. V. Lloyd-Davies, has been added to the old edition, and the illustrations here are extremely clear. This is followed by a chapter, invaluable to the practitioner, on rectal diagnosis, which includes clear accounts of proctoscopy and sigmoidoscopy as well as case-taking and record-keeping. An account of the Principles of Rectal Surgery with a description of pre- and post-operative treatment and anæsthesia conclude the general opening chapters. The rest of the book is devoted to specific lesions of the rectum and anus. While the treatment is somewhat dogmatic, it gives the impression of being the methods used regularly and successfully by one man. This is, however, preferable to a long and incoherent account of many methods where the reader is left to discriminate between them. One is somewhat surprised to find no account of the abdomino-perineal method of excision of the rectum. One also wishes that a description of the prone position for excision of the rectum had been given. This is surely much simpler that the lateral position. However, these are molehills and Mr. Gabriel is to be congratulated on his useful book.

**The Abdominal Surgery of Children.** By Sir L. BARRINGTON-WARD, K.C.V.O., Ch.M., F.R.C.S. Second Edition. Pp. xv., 333. Illustrated. London: Oxford University Press (Humphrey Milford). 1937. Price 25s.—The professi is greatly enriched when one of its members shares his singular knowledge, skill and experience on such an important topic with his lesser brethren. In this work Sir Lancelot Barrington-Ward has presented to us fully and lucidly all his ripe knowledge on the comparatively specialized field of abdominal surgery in the child. After seeing him operate and reading this book, one feels strongly that he is the best qualified among us to set forth standard surgical teaching on this subject. The book embraces the whole realm of abdominal surgery and deals fully with etiology as well as diagnosis and treatment. While mentioning what is worthy in the newer views, he is essentially cautious and sound in handling problems. He makes plain the simple truth that the mortality of appendicitis, of hypertrophic pyloric stenosis, and of intussusception can be controlled best by the early diagnosis of the general practitioner and prompt surgery. Hernia and its treatment is admirably discussed and the multitude of congenital defects of the intestine are arrayed clearly. Terminal ileitis and subphrenic abscess deserve description in the next edition. It is an invaluable book of reference for surgeons who meet but occasionally with rarities and a most admirable text-book for all who wish to learn about surgical disease in children.

**Text-book of Gastroscopy.** By N. HENNING, translated by H. W. RODGERS, F.R.C.S. Pp. xii., 86. Illustrated. London: Oxford University Press (Humphrey Milford). 1937. Price 7s. 6d.—It is only within the last few years that gastroscopy has been at all extensively practised in England. Rigid instruments are not easily introduced into the stomach; they afford but a limited view of its interior; while lack of gentleness and skill on the part of the operator or a struggle on that of the patient may easily cause serious damage to the stomach or œsophagus. Since the invention of the Wolf-Schindler flexible gastroscope, however, gastroscopy has been increasingly employed at many of our bigger medical centres. The passage of this instrument presents little more difficulty than does that of a stomach tube, and the soft rubber ball or sponge in which it terminates efficiently safeguards the patient from injury. If the stomach is empty a good view can usually be obtained so that patients are best examined after a night's fast. A preliminary washout is *not* to be recommended, as it is not easy to empty the stomach completely and the appearances of the mucous membrane after a wash are apt to be deceptive. The lesser curvature and part of the cardiac end of the stomach are not readily visible because the instrument lies in close contact with these parts of the wall of the viscus, but Dr. Rogers has invented a modification of the standard instrument whereby a rubber bag can be inflated just above the objective, thus pushing it away from the mucous membrane, and so bringing the latter into focus. With a little experience the fundus, greater curvature, pylorus, and antrum can generally be satisfactorily inspected, but much more extensive practice is required before the picture can be correctly interpreted. Gastroscopy is of great value as an additional method of diagnosis in those cases in which a test meal and radiography still leave one in doubt. By this means the reviewer has been able to determine definitely the absence of malignant changes in a doubtful ulcer, for instance, thus saving the patient from an exploratory operation. Gastroscopy has already added greatly to our knowledge of the appearances of gastritis in its various forms, and it is to be hoped that increasing familiarity with this condition may aid us in its prevention and cure. As carcinoma is commonly preceded by gastritis one can only trust that we may eventually see some diminution of this dire disease. The present volume, which is one of the well-known *Oxford Medical Publications*, is simply written, well printed and indexed and contains numerous illustrations, many of them in colour. It should prove an excellent introduction to the subject of gastroscopy.

**The Bed Bug.** British Museum (Natural History) Economic Series, No. 5. By A. W. McKenny-Hughes. Fourth Edition. Pp. iv., 19. Illustrated. 1937. Price 6d.—This is the fourth edition, and has been entirely rewritten in the light of present-day experience. The life - history, habits and means of dissemination are fully and completely described. The methods of control which are important in a pamphlet of this character are the most modern. While fumigation by HCN is most effective, the author strongly emphasizes the danger of this process if not carried out with extreme care and in charge of skilled operators. The subject-matter has been reduced without in any way detracting from its usefulness as an authoritative treatise on the subject. The lack of an index is not a material omission as the hand-book maintains a connected sequence. The previous excellent illustrations are reproduced with the addition of a fine coloured one. It can be recommended as an easily readable informative pamphlet of special interest to medical officers of health, sanitary inspectors and others whose business requires knowledge of the best means for the eradication of this pest.

**Clinical Contraception.** By G. M. Cox, M.B., B.S. Second Edition. Pp. ix., 196. Illustrated. London: William Heinemann Ltd. 1937. Price 7s. 6d.—The publication of a second edition of this text-book within so few years of the appearance of the first edition is a guarantee that the practitioner will find in it practical and up-to-date information on modern contraceptive methods. There is no doubt that a wider knowledge of the clinical uses of chemical contraceptives and of mechanical appliances for birth control is now expected of the medical profession, and there are few British medical authors better qualified through practical experience than Dr. Cox to supply the information which is required by doctors in general practice. The physiological and medical aspects of the subject are dealt with in a scientific manner, and the pros and cons of the different methods are fully set forth. Chapter III is devoted to a comparison of the different values of chemical contraceptives, and to the methods of their application; reference is also made to recent research work on the spermicidal value of the chemical contraceptives in most general use. This chapter contains information of particular value to the practitioner. In the final chapters the various contraceptive methods are compared, and useful information is given about the powers of Local Authorities in England and Wales to provide facilities for advice and instruction in methods of contraception. This book can be recommended to those seeking information on this subject.

**The Highveld Climate.**—This small pamphlet, issued by the Publicity Bureau of the Union of South Africa, supplies a series of meteorological data such as rainfall, annual sunshine hours, temperatures, humidity and other climatic details, which pertain to the high plateau constituting the interior of the more northern portions of South Africa. These observations make interesting reading and supply a rational basis for the claims of this area as a health resort.

**First Aid to the Injured and Sick.** Edited by F. C. NICHOLS, M.C., M.B., Ch.B., M.R.C.S., L.R.C.P., L.D.S. Sixteenth Edition. Pp. xii., 318. Bristol: John Wright & Sons, Ltd. 1937. Price 2s. 6d. net.—The sixteenth edition of Warwick and Tunstall's *First Aid* differs from the previous editions in containing a full and up-to-date account of gas poisoning in warfare. The particular points which have been expanded or added since the fifteenth edition are: fuller details of treatment, new sections on detection of various types of gas, new sections on protection of houses and buildings (admirably illustrated) and on protection of the body from blistering gases and liquids. There is also a new and adequate account of Air Raid Precaution Organizations, with full description of First Aid and Decontamination Posts. The value of this favourite manual of first aid is greatly increased by these additions.

